# How Suppressed Anger Can Become an Illness: A Qualitative Systematic Review of the Experiences and Perspectives of Hwabyung Patients in Korea

**DOI:** 10.3389/fpsyt.2021.637029

**Published:** 2021-05-28

**Authors:** Hyo-Weon Suh, Ki-Beom Lee, Sun-Yong Chung, Minjung Park, Bo-Hyoung Jang, Jong Woo Kim

**Affiliations:** ^1^Department of Neuropsychiatry, College of Korean Medicine, Kyung Hee University, Seoul, South Korea; ^2^Medical Unit of Capital Mechanized Infantry Division, Republic of Korea Army, Gapyeong County, Gyeonggi Province, South Korea; ^3^Department of Neuropsychiatry, Kyung Hee University Korean Medicine Hospital at Gangdong, Seoul, South Korea; ^4^National Agency for Development of Innovative Technologies in Korean Medicine, National Institute for Korean Medicine Development, Seoul, South Korea; ^5^Department of Preventive Medicine, College of Korean Medicine, Kyung Hee University, Seoul, South Korea

**Keywords:** culture, anger, hwabyung, systematic review, qualitative research, meta-aggregation

## Abstract

**Background:** In the clinical field, anger has generally been studied in terms of aggressive behavior. However, in Asians, anger suppression is more common than anger expression. Hwabyung is a culture-related anger syndrome in Korea and is known to occur due to the continued repression of anger. Investigating Hwabyung should lead to a better understanding of the multiple dimensions of anger. To explore Hwabyung patients' experiences and perspectives, a meta-aggregation approach was used to conduct a systematic review and a qualitative synthesis.

**Methods:** A systematic search was conducted in MEDLINE/PubMed, EMBASE, Allied and Complementary Medicine Database (AMED), Cumulative Index to Nursing and Allied Health Literature (CINAHL), PsycARTICLES, and four Korean databases [Korean Medical Database (KMbase), Korean Studies Information Service System (KISS), National Digital Science Library (NDSL), and Oriental Medicine Advanced Searching Integrated System (OASIS)] in September 2020. Studies were included if they collected and analyzed qualitative data from Hwabyung patients. Qualitative research findings on the experiences and perspectives of Hwabyung patients in Korea were critically appraised and synthesized using the Joanna Briggs Institute methodology.

**Results:** Seven eligible studies were included. The findings from those studies (i.e., theme or subtheme of qualitative research) were aggregated into categories (a group of similar findings) and synthesized findings (a group of categorized findings). Ultimately, 116 findings were aggregated into 15 categories. Finally, four synthesized findings were derived from the 15 categories: (i) anger arousal, (ii) blame, (iii) uncontrollable physical and emotional symptoms, and (iv) compromise and temporary coping.

**Conclusions:** Patients with Hwabyung experience chronic anger through the complex cognitive processes involved in blame. Hwabyung negatively affects patients' physical, psychological, and social functions. Because Hwabyung patients feel as if they are losing control, due to emotional dysregulation and physical symptoms, professional support should be provided to facilitate their coping strategies. Further studies on Hwabyung can serve as a new model of pathological anger.

## Introduction

The American Psychiatric Association (APA) has focused on expressive anger and aggression in the Diagnostic and Statistical Manual of Mental Disorders (DSM). Western culture generally encourages the expression of emotions rather than suppression. To date, most studies on suppression have concluded that suppression negatively affects social outcomes (1). Although the suppression of emotion is viewed as dysfunctional, anger suppression has received relatively little academic attention. To address this gap, it is necessary to study the pathological form of anger suppression.

Compared to Western culture, Asian values, such as collectivism, harmony, and interdependence, encourage the suppression of negative emotions to maintain or strengthen social networks (2, 3). As Min (4), a Korean psychiatrist, pointed out, Koreans hold collective memories of historical tragedies, which include repeated foreign invasions, Japanese colonization, and the Korean War. To overcome the emotional crises caused by such memories, Koreans may suppress their emotions and use this suppression as a long-term strategy against overwhelming external violence. According to Min, this tendency is also maintained even when Koreans are faced with interpersonal problems. However, the suppression of emotions due to social pressure works differently for men and women. Korea's patriarchal system urges married women to suppress their emotions more than men and to become a “Wise Mother and Good Wife” (5, 6). Korean women are faced with dual and contradictory social demands to obey their husbands and to be strong for their children (7). In the Korean cultural structure and context, most of the intentionality of women's emotions is distorted or denied, and their anger is not recognized but rather excessively suppressed to the point of illness (8).

Hwabyung is a unique phenomenon observed among Koreans that occurs because of the suppression of anger over a long period of time. Hwabyung was first reported in the American Journal of Psychiatry in 1983 by Lin (9), and it was listed in the “culture-bound syndrome” category of the DSM-IV in 1994 (10). In Korea, Hwabyung is widely recognized as a traditional disease and is classified as U22.2 in the Korean version of the International Classification of Diseases (ICD). Epidemiological studies have reported that the prevalence of Hwabyung in Korea is 4.2–13.3% (11–14). According to data from the Korean Health Insurance Review and Assessment Service, the number of patients who used healthcare services to treat Hwabyung reached 14,064 in 2019, accounting for 0.03% of the total population.

The term Hwabyung literally translates as “fire-illness” in English, because physical symptoms of the disorder include a heat sensation, as if one has flames in their body; the feeling of something pushing up in one's chest; respiratory stuffiness; and dry mouth (15). However, psychiatric researchers usually use the term “anger syndrome” when referring to the disorder in English because its most prominent cause and manifestation is anger (16). Hwabyung develops when anger or feelings of unfairness are suppressed and accumulated after exposure to stressful life events (4). Middle-aged Korean women generally perceive vulnerable situations, lowered self-esteem, and negative life events as the primary causes of Hwabyung (17). The common etiological life events of Hwabyung patients include conflicts between them and their spouses or in-laws and economic hardship (18).

A multidisciplinary team consisting of psychiatrists, doctors of traditional Korean medicine, and psychologists developed and validated the standardized diagnostic criteria for Hwabyung (19). The criteria include both physical symptoms reminiscent of being on fire and psychological symptoms related to anger. To receive a diagnosis of Hwabyung, these symptoms must cause a significant decline in functions in social, occupational, or other important areas, and there must be previous causal stress associated with the symptoms. The symptoms must also not be a result of substance abuse or other medical conditions. In 2020, the Korean Society of Oriental Neuropsychiatry, composed of doctors of traditional Korean medicine, suggested new specifiers for Hwabyung as follows: rapid-onset, acute, chronic, explosive type, and suppressive type (20).

One open-label clinical trial examined paroxetine, a selective serotonin reuptake inhibitor, in Hwabyung patients. Paroxetine decreased patients' total scores on the Hamilton Depression Rating Scale (HDRS), the Hwabyung scale, and the State-Trait Anger Expression Inventory (STAXI), but it did not effectively decrease pathognomonic symptoms such as “Haan” [a mixed feeling of sorrow, regret, sadness, and depression, or an aggressive feeling of hatred and revenge (15, 21)], guilt, hostility, and the sensation of a mass in the throat or epigastric region (22). A randomized controlled trial comparing the herbal medicine “Bunsimgi-eum” and placebo was also conducted, but it did not show significant effects (23). These results imply that the biochemical basis of Hwabyung is still incomplete. Several researchers have suggested various psychological and ecological models for Hwabyung (24), and Suh (25) extensively reviewed articles on Hwabyung, concluding that it is necessary to listen to patients' narratives to treat Hwabyung.

Accordingly, this study aimed to synthesize the evidence from qualitative studies of Hwabyung in order to explore the characteristics of the disorder and fully understand patients' experiences and perspectives.

## Methods

### Search Strategy

A systematic search was conducted in MEDLINE/PubMed, EMBASE, Allied and Complementary Medicine Database (AMED), Cumulative Index to Nursing and Allied Health Literature (CINAHL), PsycARTICLES, and four Korean databases [Korean Medical Database (KMbase), Korean Studies Information Service System (KISS), National Digital Science Library (NDSL), and Oriental Medicine Advanced Searching Integrated System (OASIS)] on September 7, 2020. The search terms used were “Hwabyung,” “anger disorder,” “anger syndrome,” and “fire-illness.” There were no limitations to the search in terms of language and publication date. Further details of the search protocol are provided in Supplementary Table 1.

### Study Selection

The inclusion and exclusion criteria were developed and organized using the SPIDER (Sample, Phenomenon of Interest, Design, Evaluation, Research type) tool (26). The samples were Hwabyung patients diagnosed solely based on the judgment of a doctor or with standardized diagnostic instruments. Studies on individuals who believed they had the disorder but had not been officially diagnosed were excluded. Severe psychiatric disorders, such as bipolar disorder, delusion disorder, schizophrenia, and intellectual disabilities, and critically unstable medical conditions, such as cancer, cardiovascular disease, and liver or kidney diseases, were also excluded. The phenomena of interest were the experiences of Hwabyung and the perspectives of patients suffering from the disorder. Studies were included if they were designed to collect data through surveys, interviews, or observations, and data were analyzed using qualitative research methodologies such as ethnography, grounded theory, phenomenology, and consensual qualitative research. Both qualitative and mixed-methods studies were included. However, studies with unsupported results or no themes were excluded. Quantitative studies were also excluded.

Two researchers (HWS and KBL) independently screened and excluded studies that were not qualitative or were not related to Hwabyung, through evaluation of their titles and abstracts. The full text of the potential articles was then retrieved to consider eligibility. Disagreements were resolved by consensus with a third-party reviewer (JWK).

### Quality Assessment

Methodological quality was assessed using the Joanna Briggs Institute (JBI) critical appraisal checklist for qualitative research, which is a 10-item standardized critical appraisal instrument (27). Five items (numbers 2, 3, 4, 6, and 7) assess the quality of the methodology and are used to determine the ConQual-Dependability ranking (28). The other five items assess congruency from a philosophical perspective, methodology and results interpretation, presentation of participants, ethics, and the conclusion.

The ConQual-Credibility ranking represents the congruency between the authors' interpretation and the study data. Each finding was assessed as “Unequivocal (U),” “Credible (C),” or “Not supported (N)” based on the extent to which they were supported by illustrations from participants' voices. Unequivocal findings are supported by evidence that is beyond reasonable doubt that is not open to challenge. Credible findings are interpretive and, therefore, open to challenges. Findings that are classified as “Not supported” cannot be supported by the data, and therefore, they are not included in the meta-aggregation. Two researchers (HWS and KBL) independently assessed each study. Discrepancies are also discussed.

### Data Extraction and Data Synthesis Methods

We used the JBI method to extract and synthesize review findings (27). To determine the characteristics of the included studies, we extracted the year of publication, first author, country, study objectives, characteristics and number of participants, diagnostic criteria used for Hwabyung, methodologies and methods, and whether interventions had been applied. To synthesize the qualitative evidence, we extracted themes, subthemes, and supportive quotations from the original studies. If a study qualitatively investigated the effects of an intervention, we only extracted the findings related to the phenomena of interest and excluded the findings related to the effects or responses to treatment.

Data were extracted by the first author (HWS) and verified by another researcher (KBL). To clarify the meaning of the extracted findings, the first author (HWS) thoroughly reviewed all findings and quotations. The first author's aggregated findings were categorized by similarities, and then four researchers (SYC, MP, BHJ, and JWK) reached a consensus on the results. Finally, the synthesized findings were drawn from the resulting meta-aggregation of categories.

### Confidence of Synthesized Findings

The level of confidence of the synthesized findings was assessed and evaluated using the ConQual approach (28) which is based on dependability and credibility. Dependability refers to the consistency and stability of qualitative findings, which corresponds to reliability in quantitative studies. Contrastingly, credibility is the extent to which qualitative findings represent the truth, which corresponds to internal validity in quantitative studies (29). At the start of the ConQual assessment, qualitative research starts at “High,” and expert opinion starts at “Low.” Downgrading of dependability occurs when the included studies do not meet at least four of the five criteria for dependability, and downgrading for credibility occurs when there is a mixture of unequivocal and equivocal findings. The confidence of estimates of the synthesized findings for both dependability and credibility leads to an overall ranking for each synthesized finding, which ranges from “high” to “very low” (“high,” “moderate,” “low,” and “very low”).

## Results

### Study and Participant Characteristics

**Figure 1:**
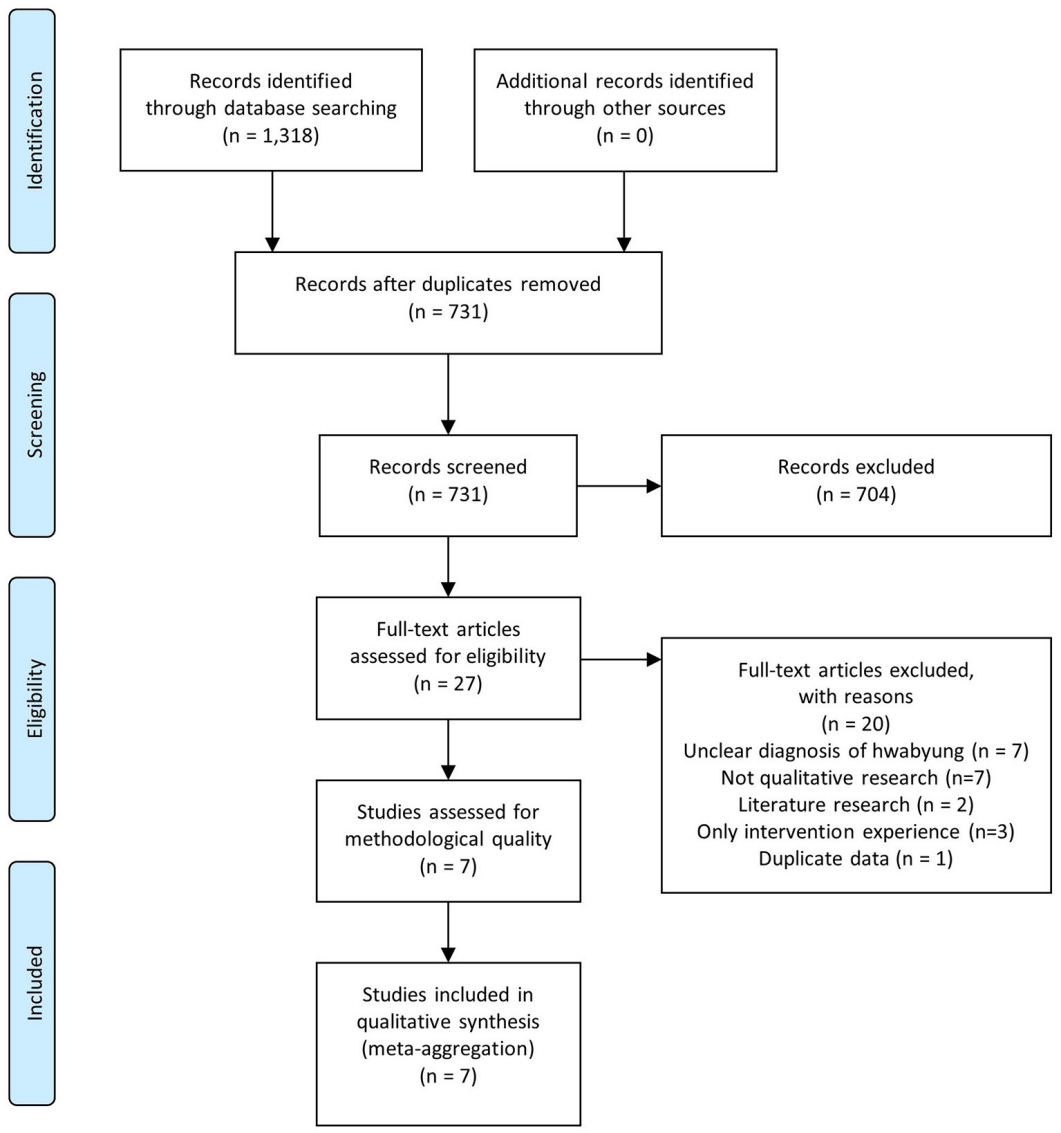
Preferred Reporting Items for Systematic Reviews and Meta-Analyses (PRISMA) flow chart.

An electronic search yielded 1,318 articles. A total of 731 potential articles remained after the duplicates were removed. After screening the titles and abstracts, 27 articles were selected for full-text analysis. Finally, seven studies (30–36) were included and synthesized in this review (Figure 1). The overall characteristics and extracted findings of the included studies are summarized in Tables 1 and 2, respectively.

**Table 1:** Characteristics of included studies.

**No**.	**Study**	**Setting**		**Objective**	**Participants (*n*)**	**Diagnosis criteria**	**Methodology**	**Methods**
		**Country**	**1 = stand-alone;** **2 = alongside an intervention**					
1	Park et al. (30)	Korea	1	To reexamine the conceptual structure of Hwabyung through a qualitative analysis of data collected from interviews of middle-aged Korean women with Hwabyung	Women diagnosed with Hwabyung, aged 40 to 65 (*n* = 6)	Diagnosed by a doctor of traditional Korean medicine	Content analysis	An open-ended in-depth interview
2	Chae (31)	Korea	1	To comprehensively and deeply investigate the experiences of middle-aged women with Hwabyung, examining the role of culture and helping those women to reflect on themselves and to seek a better life, adding to the basic nursing data	Middle-aged women with Hwabyung (*n* = 5)	Diagnostic criteria developed by a psychiatrist and developed by a doctor of traditional Korean medicine	Ethnography	In-depth interview and participant observation
3	Choi (32)	Korea	1	To investigate the daily experience of women with Hwabyung to develop a formal theory that explains the lives of middle-aged women with Hwabyung, and to provide greater understanding of middle-aged women with the disorder in hopes of improving nursing practice for these patients	Women who were diagnosed with Hwabyung (*n* = 11)	HBDIS	Grounded theory	In-depth interview and participant observation
4	Kim (33)	Korea	2	To explore the lives of women who experience Hwabyung with upper limb arthropathy, and to deepen the understanding of nursing intervention in these patients	Women with Hwabyung and upper limb arthropathy (*n* = 9)	HBDIS	Ethnography	In-depth interview
5	Song (34)	Korea	1	To describe the progress of and recovery from Hwabyung and the influencing factors	Participants who suspected they had Hwabyung, including actual Hwabyung patients (*n* = 6) and non-Hwabyung patients (*n* = 2) (Participants were all women)	SCI for the modified HBDIS	CQR	In-depth interview
6	Park et al. (35)	Korea	1	To understand the daily lives and values of middle-aged women with Hwabyung, and to focus on how they reorganized their lives to escape their crisis and how they continue moving forward	Middle-aged women with Hwabyung (*n* = 5) and family members (*n* = 2)	Questionnaire for differentiating Hwabyung	Ethnography	In-depth interview and participant observation
7	Suh (36)	Korea	2	To describe the development of Hwabyung, the experiences of the participants in an EFT group program, and perspectives on prognosis of the disorder	Hwabyung patients who participated in a 4-week EFT group treatment program (*n* = 5) (Patients were all women)	SCI for the modified HBDIS	Phenomenology	In-depth interview

*CQR, Consensual qualitative research; EFT, emotional freedom techniques; HBDIS, Hwa-Byung Diagnostic Interview Schedule; SCI, Structured Clinical Interview*.

**Table 2:** Findings of included studies.

**Study**	**Dependability**	**Numbered findings**	**Credibility**
Park et al. (30)	3	1 Women themselves and life situations: Strong commitment to traditional values	U
		2. Women themselves and life situations: Their own quick-tempered personality	U
		3. Women themselves and life situations: Conflicted marital relationship	U
		4. Women themselves and life situations: A hard life	U
		5. Women themselves and life situations: An unhappy life	U
		6. Nature of experiences/responses: Endurance (forbearance)	U
		7. Nature of experiences/responses: Feelings of victimization or mortification	U
		8. Nature of experiences/responses: Anger	U
		9. Nature of experiences/responses: Deep sorrow (“Haan”)	U
		10. Symptoms	U
Chae (31)	5	11. Perfect and timid personality: Passive disposition	U
		12. Perfect and timid personality: Imperfect one	U
		13. Perfect and timid personality: Compulsory one	U
		14. The collapse of the family: Violent home	U
		15. The collapse of the family: Family breakup	N
		16. The collapse of the family: Financial problems	N
		17. Socio-cultural problem: Shunned and mistreated by neighbors	U
		18. Abandoned as a criminal: Lost	C
		19. Abandoned as a criminal: Sinned	U
		20. Abandoned as a criminal: Humble status	N
		21. Abandoned as a criminal: Isolation	C
		22. Falling into the depths of agony: Chased and uncomfortable	U
		23. Falling into the depths of agony: Stubbornness and exhaustion	C
		24. Falling into the depths of agony: Feelings of being small and weak, and having no confidence to handle daily life	N
		25. Falling into the depths of agony: Tantrums	U
		26. Falling into the depths of agony: Lamentation and self-abandonment	U
		27. Self-restoration after cutting off unwanted ties: Cutting a connection and cherishing new hope	U
		28. Self-restoration after cutting off unwanted ties: Peace in extreme conditions	U
		29. Self-restoration after cutting off unwanted ties: Self-discovery	U
Choi (32)	4	30. Left in loneliness	U
		31. Continual suffering	U
		32. Frustration over the failures of one's children	U
		33. Sensation of one's soul burning and singed	U
		34. Thrusted into bondage	U
		35. Awareness of Hwabyung	C
		36. Stumbling blocks	U
		37. Buttresses	U
		38. Caring for the body and spirit (used twice)	U
		39. Seeking resolutions	U
		40. Giving up the whole self	U
		41. Suppressing rage	U
		42. Counteracting with fire	U
		43. Releasing the heart	U
		44. Changing interests	U
		45. Hanging onto faith	U
		46. Reviving hope	U
		47. Body left as an empty shell	U
		48. Intense anguish (“Haan”)	U
		49. Immersed in regret	U
		50. Transcendence	U
		51. Journey of self-discovery	U
Kim (33)	4	52. External causes: Patricentric family system	C
		53. External causes: Husband having an extramarital affair	U
		54. External causes: Cruel treatment by husband's family	U
		55. External causes: Financial failure	U
		56. The discord between husband and wife: Discontinued communication	U
		57. The discord between husband and wife: Lack of understanding	U
		58. The discord between husband and wife: Becoming unfeeling, heartless, or unsympathetic toward husband	U
		59. The discord between husband and wife: Not related to lack of sexual relations	U
		60. Coexisting emotions: The feeling of anger	U
		61. Coexisting emotions: The desire to escape	U
		62. Coexisting emotions: The sense of nihility	U
		63. Coexisting emotions: The attempt to forgive	U
Song (34)	3	64. Insights into Hwabyung: Insights at onset of Hwabyung	U
		65. Insights into Hwabyung: Current insights	U
		66. Cognition regarding recovery: Passive cognition	U
		67. Cognition regarding recovery: Active cognition	U
		68. Environmental changes: End of stressful events	U
		69. Environmental changes: Reduction in external stimuli	U
		70. Environmental changes: Life changes	U
		71. Changes in physical symptoms: Reduction in symptoms	U
		72. Changes in physical symptoms: Becoming more sensitive to symptoms	U
		73. Changes in physical symptoms: Control of physical symptoms	U
		74. Cognitive changes: Reduction in rumination	U
		75. Cognitive changes: Changes in cognition and coping with stress	U
		76. Cognitive changes: Relief in re-experience	U
		77. Cognitive changes: Improved coping skills	U
		78. Emotional changes: Positive emotions	U
		79. Emotional changes: Relief in anger	U
		80. Emotional changes: Decreased impulsivity	U
		81. Emotional changes: Emotional stability	U
		82. Emotional changes: More expressions of anger	U
		83. Change of attitude toward others: Improvements in relationships	U
		84. Change of attitude toward others: Increased understanding of others	U
		85. Change of attitude toward others: Decrease in conflicts	U
		86. Change of attitude toward others: Practicing loving kindness	U
		87. Change of attitude toward others: Being generous	U
		88. Recovery factors: Reduction in stimuli	U
		89. Recovery factors: Social support, interpersonal relationships	U
		90. Recovery factors: Exercise	U
		91. Recovery factors: Treatment	N
		92. Recovery factors: Control of thought involvement and repetition	U
		93. Recovery factors: Development of a positive self-image	U
		94. Recovery factors: Better understanding of others	U
		95. Recovery factors: Making a life for oneself	U
		96. Aggravation factors: Increased stress	U
		97. Aggravation factors: Obsessing over past events	U
Park et al. (35)	4	98. Changing their perspectives on life: Changing of perspectives	U
		99. Changing their perspectives on life: Finding hope through their children	U
		100. Renewing family relationships: Repairing relationships	U
		101. Renewing family relationships: Strengthening family relationships	U
		102. Rearrangement of family roles: Managing to cope with their own roles	U
		103. Rearrangement of family roles: Taking on the husband's role	U
		104. Rearrangement of family roles: Reinforcement of individual capacities	U
Suh (36)	5	105. Pattern of Hwabyung: Repressive type	U
		106. Pattern of Hwabyung: Volatile type	U
		107. Persistence factors: Repressive personality traits	U
		108. Persistence factors: Volatile personality traits	U
		109. Persistence factors: Life Stress	U
		110. Persistence factors: Rumination/retrospection	U
		111. Persistence factors: Absence of emotional outlet	U
		112. Achievements and failures of attempts: Attempt to communicate	U
		113. Achievements and failures of attempts: Counseling	U
		114. Achievements and failures of attempts: Medication	U
		115. Achievements and failures of attempts: Traditional Korean medicine treatment	U
		116. Achievements and failures of attempts: No experience of treatment	U
		117. Recognition of the future: It's up to me	U
		118. Recognition of the future: I cannot escape	U
		119. Future coping strategies: Emotional control	U
		120. Future coping strategies: Avoiding conflict	U
		121. Future coping strategies: Seeking professional treatment	U

This review included 51 participants. Most of the studies (30–33, 36) included only Hwabyung patients. Song (34) included two participants who believed they had Hwabyung but did not meet the diagnostic criteria. Park et al. (35) included two family members of patients. All participants with complaints of Hwabyung symptoms (clinical or non-clinical) were women.

Participant eligibility was primarily determined using the Hwa-Byung Diagnostic Interview Schedule (HBDIS) (19) or the structured clinical interview for the modified HBDIS (37) in four studies (32–34, 36). Chae (31) used two items: unpublished diagnostic criteria provided by a psychiatrist and invalidated diagnostic criteria reported by a doctor in traditional Korean medicine (38). Park et al. (35) used a questionnaire to determine which patients had Hwabyung (39). Park et al. (30) included patients who were diagnosed by a doctor in traditional Korean medicine, without any presentation of standardized diagnostic criteria.

All included qualitative studies were conducted in Korea. Five (30–32, 34, 35) of the seven studies did not include interventions. Kim (33) recruited participants among patients who had visited a doctor of traditional Korean medicine seeking treatment for upper limb arthropathy. A qualitative research was conducted along with treatment. Suh (36) conducted a qualitative research as part of a clinical trial (40) and included those who had completed the trial.

### Quality of Included Studies

All included studies met at least seven of the ten criteria of the JBI critical appraisal checklist for qualitative research (27): Chae (31) and Suh (36) met all the criteria. Choi (32) and Park et al. (35) met nine criteria. Kim (33) and Song (34) met eight criteria, and Park et al. (30) met seven criteria. Among the items, Q1 was met by five studies (31, 32, 34–36), Q6 by three studies (31, 32, 36), and Q7 by four studies (31, 33, 35, 36). The remaining criteria were satisfied by all the studies. Overall, the methodological quality of the studies was good because they met the majority of the predetermined criteria. No studies were excluded from the synthesis because they were all found to be of good quality (Table 3).

**Table 3:** Quality assessment of included studies.

**Study**	**Q1**	**Q2**	**Q3**	**Q4**	**Q5**	**Q6**	**Q7**	**Q8**	**Q9**	**Q10**
Park et al. (30)	U	Y	Y	Y	Y	N	U	Y	Y	Y
Chae (31)	Y	Y	Y	Y	Y	Y	Y	Y	Y	Y
Choi (32)	Y	Y	Y	Y	Y	Y	U	Y	Y	Y
Kim (33)	U	Y	Y	Y	Y	U	Y	Y	Y	Y
Song (34)	Y	Y	Y	Y	Y	U	N	Y	Y	Y
Park et al. (35)	Y	Y	Y	Y	Y	U	Y	Y	Y	Y
Suh (36)	Y	Y	Y	Y	Y	Y	Y	Y	Y	Y
Total %	71	100	100	100	100	43	57	100	100	100

*Y, yes; U, unclear; N, no; Joanna Briggs Institute (JBI) Critical Appraisal Checklist for Qualitative Research*.

*Q1, Is there congruity between the stated philosophical perspective and the research methodology?*

*Q2, Is there congruity between the research methodology and the research question or objectives?*

*Q3, Is there congruity between the research methodology and the methods used to collect data?*

*Q4, Is there congruity between the research methodology and the representation and analysis of data?*

*Q5, Is there congruity between the research methodology and the interpretation of results?*

*Q6, Is there a statement locating the researcher culturally or theoretically?*

*Q7, Is the influence of the researcher on the research, and vice-versa, addressed?*

*Q8, Are participants, and their voices, adequately represented?*

*Q9, Is the research ethical according to current criteria or, for recent studies, is there evidence of ethical approval by an appropriate body?*

*Q10, Do the conclusions drawn in the research report flow from the analysis or interpretation of the data?*

### Summary of Findings and ConQual Assessment

One hundred twenty-one findings were extracted from seven studies. One hundred and twelve findings were appraised as being unequivocal, and four were appraised as credible. Five findings were unsupported and excluded. After exclusion of the unsupported findings, 116 extracted findings were aggregated into 15 categories. The four synthesized findings were derived from the 15 categories: (i) anger arousal, (ii) blame, (iii) uncontrollable physical and emotional symptoms, and (iv) compromise and temporary coping. Illustrative quotations are presented in Supplementary Table 2.

Among the synthesized findings, blame, uncontrollable physical and emotional symptoms, and compromise and temporary coping received moderate ConQual scores, whereas anger arousal received low ConQual scores (Table 4).

**Table 4:** Summary of findings and ConQual assessment.

**Synthesized findings**	**Type of research**	**Dependability**	**Credibility**	**ConQual score**	**Comments**
Anger arousal	Qualitative	Moderate (downgraded 1 level)	Moderate (downgraded 1 level)	Low	Dependability downgraded: Of 6 primary studies, 2 studies addressed all 5 dependability questions, 2 studies addressed 4 dependability questions, and 2 studies addressed 3 dependability questions. Credibility downgraded due to mix of U and C findings (19U+4C)
Blame	Qualitative	Moderate (downgraded 1 level)	High (no change)	Moderate	Dependability downgraded: Of 7 primary studies, 2 studies addressed all 5 dependability questions, 3 studies addressed 4 dependability questions, and 2 studies addressed 3 dependability questions. Twenty-four findings were included; all were unequivocal.
Uncontrollable physical and emotional symptoms	Qualitative	Moderate (downgraded 1 level)	High (no change)	Moderate	Dependability downgraded: Of 5 primary studies, 2 studies addressed all 5 dependability questions, 1 study addressed 4 dependability questions, and 2 studies addressed 3 dependability questions. Twenty-three findings were included; all were unequivocal.
Compromise and temporary coping	Qualitative	Moderate (downgraded 1 level)	High (no change)	Moderate	Dependability downgraded: Of 6 primary studies, 2 studies addressed all 5 dependability questions, 3 studies addressed 4 dependability questions, and 1 study addressed 3 dependability questions. Forty-seven findings were included; all were unequivocal.

*C, credible; U, Unequivocal*.

### Synthesized Finding 1: Anger Arousal

**Figure 2:**
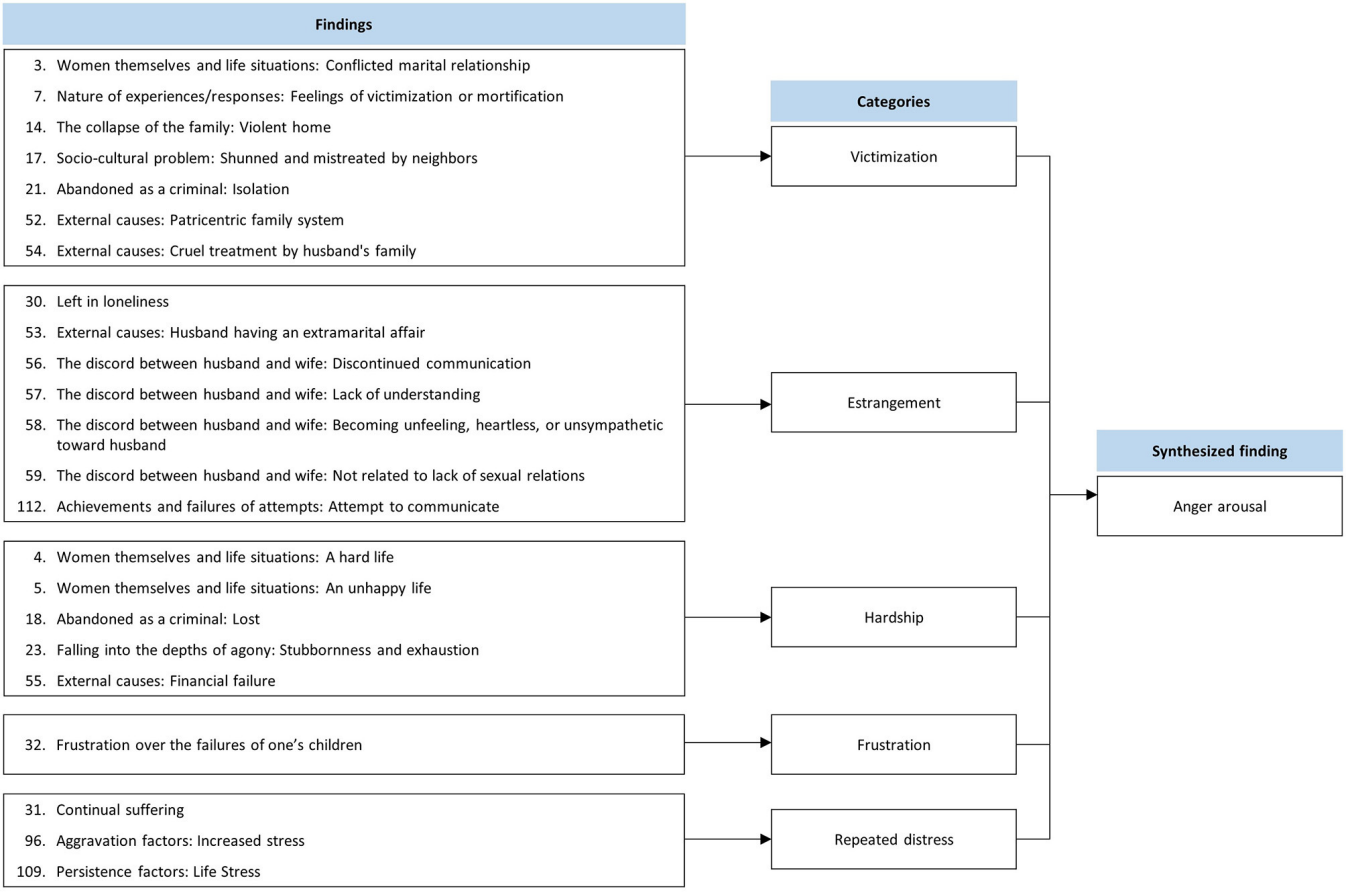
Synthesized finding 1: Anger arousal.

This synthesis was based on 23 findings aggregated into five categories (Figure 2). Hwabyung patients experienced frequent anger arousal induced by repeated and chronic life stress. Specific events were highly associated with interpersonal problems and involved physical or psychological violence, marital conflicts, financial issues, and frustration regarding their children.

#### Category 1: Victimization

Hwabyung patients reported that they were beaten and mistreated by their husbands or were in constant conflict with their in-laws (30, 31, 33). Although they had worked hard and performed their best for their families, they were not rewarded. Thus, they felt victimized and mortified (30). In some cases, Hwabyung patients were bullied by neighbors, so they felt lonely and unjust (31).

#### Category 2: Estrangement

Hwabyung patients try to communicate with their husbands but fail and eventually stop trying (32, 33, 36). Whenever they would make a claim, the husband would insult them or harshly refute the claim (32, 33). Some patients reported that their relationships with their spouses lacked understanding and affection, and that there was no communication between them (33). There have also been reports of husbands having extramarital affairs. The patients condemned their husbands for the affairs because they had not rejected their husbands sexually (33).

#### Category 3: Hardship

Most Hwabyung patients encounter overwhelming life obstacles, such as poverty (30, 31, 33). Sometimes, the patients, instead of their husbands, were responsible for earning money (30). Because of these hardships, they felt tired and unhappy (30, 31).

#### Category 4: Frustration

Some Hwabyung patients experienced frustration over disappointments regarding their children. For example, their children's academic or career failures would upset them. Besides disappointment, heartbreak over their children's illnesses also causes emotional suffering (32).

#### Category 5: Repeated and Chronic Stress

Hwabyung patients reported that stressful life events frequently provoked anger (32, 34, 36).

### Synthesized Finding 2: Blame

**Figure 3:**
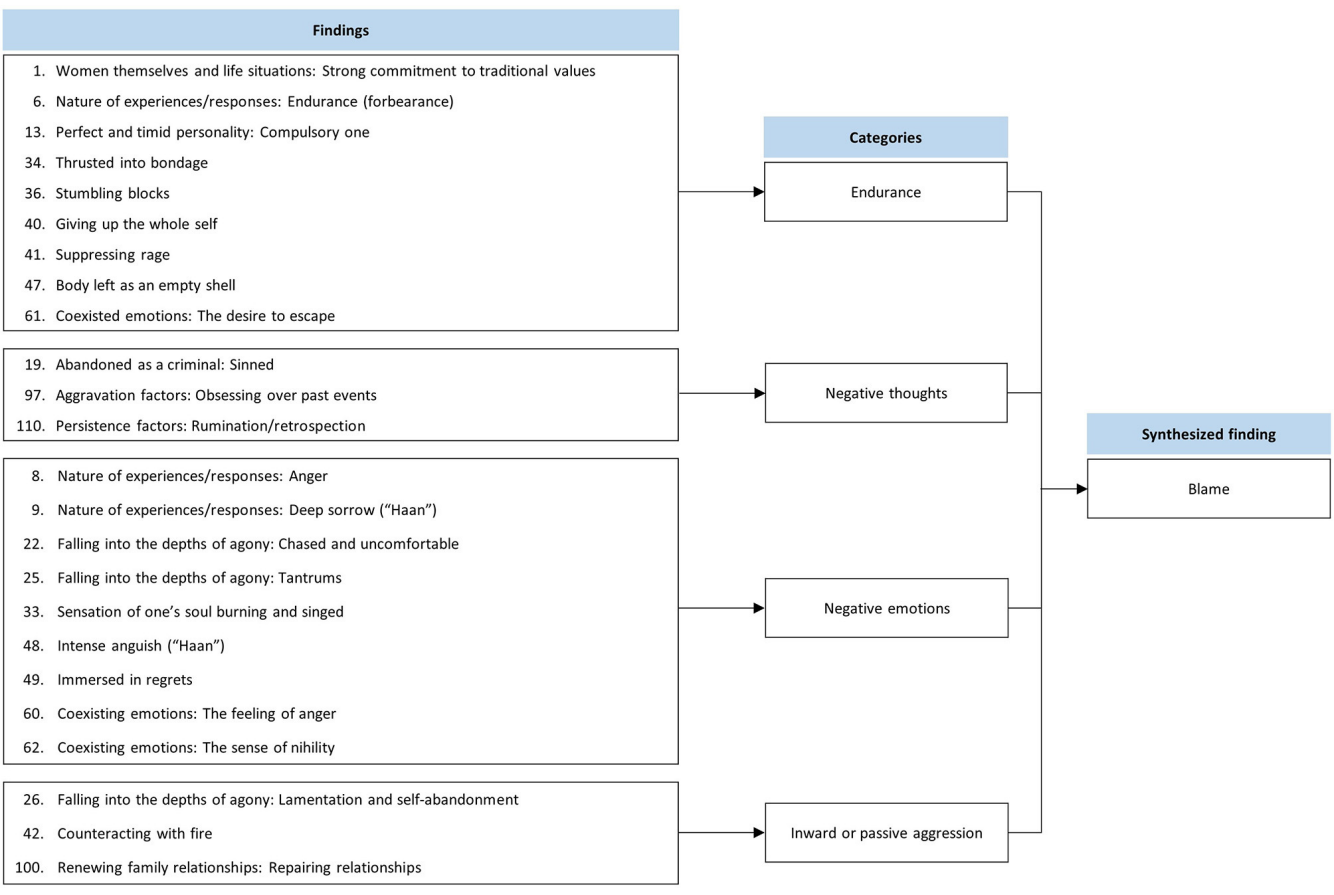
Synthesized finding 2: Blame.

This synthesis was based on 24 findings aggregated into four categories (Figure 3). Hwabyung patients suffered from abusive or uncooperative family environments, but they did not leave their families for many reasons. Instead, they felt that they faced a dilemma. During stressful situations, patients attempted to cope, suppressed anger, and sacrificed themselves. However, they still experienced a sense of helplessness and were preoccupied with negative thoughts and self-blame. They were overwhelmed by negative emotions toward themselves, as well as others who were the source of their stress. They undermined social networks through self-abandonment and passive aggressive behaviors.

#### Category 1: Endurance

Hwabyung patients had the desire to escape from reality, but they did not (33). There were two main reasons why these patients persevered: First, many believed their destinies were inevitable and that it was their duty to uphold the values of their culture (30, 32). Although the main cause of stress for many of the patients was their husbands, they had a compulsory tendency to follow Korea's patriarchal system (31). They believed that they were unable to change their situation or society, so they obeyed their husbands and maintained their families instead of getting divorced (30). Second, one of the primary goals was to raise their children well. Therefore, they sacrificed their own happiness for their children. They devoted themselves to their children and suppressed their anger, even to the point of illness (32).

#### Category 2: Negative Thoughts

Hwabyung patients had negative thought patterns, such as rumination and personalization. The patients repeatedly focused on the same thoughts related to the past. These thoughts usually trigger negative emotions and other or self-blame (34, 36). In some cases, the patient had an irrational belief that all her bad experiences were her own fault (31).

#### Category 3: Negative Emotions

Hwabyung patients experienced various negative emotions (30, 32, 33), including anger and nervousness (30, 31, 33). When the patients looked back on their lives, they expressed more complex emotions, such as regret, a sense of nihility, deep sorrow or grief (“Haan”), and painful feelings, such as burning in the soul (30–33).

#### Category 4: Inward or Passive Aggression

Hwabyung patients engage in inappropriate behaviors that weaken their interpersonal relationships (particularly with their spouses or neighbors) (31, 32, 35). Some patients neglect their self-care and withdraw from social activities (31). Other patients ignored their husbands out of revenge (believing that the husband had not treated them fairly) or to avoid conflicts (32, 35).

### Synthesized Finding 3: Uncontrollable Physical and Emotional Symptoms

**Figure 4:**
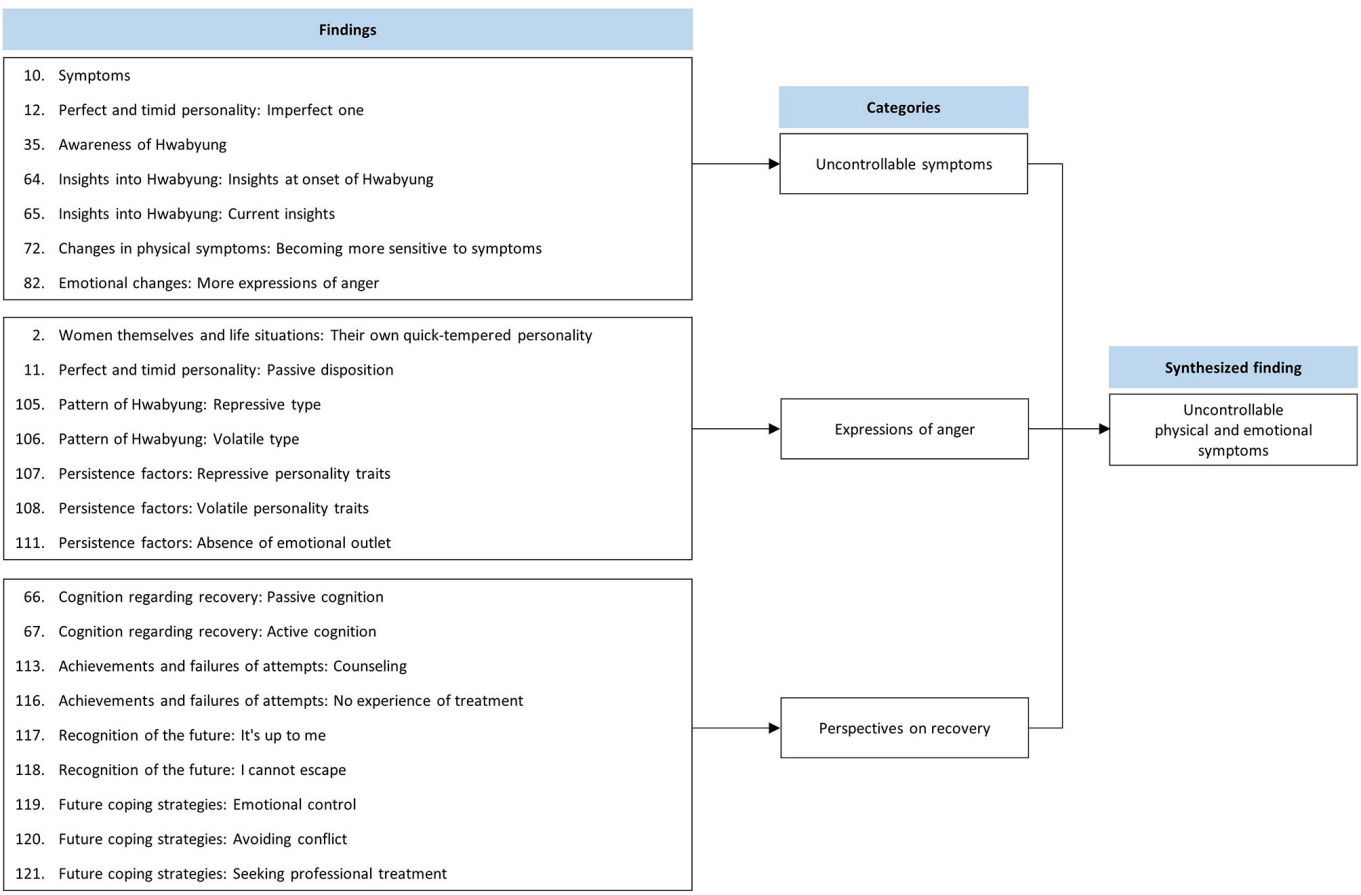
Synthesized finding 3: Uncontrollable physical and emotional symptoms.

This synthesis was based on 23 findings aggregated into three categories (Figure 4). Hwabyung patients experience uncontrollable physical symptoms and sudden rushes of anger and thoughts. They could not effectively regulate their anger and instead had emotional outbursts or suppressed their feelings. Patients also showed passive and limiting attitudes toward recovery attempts.

#### Category 1: Uncontrollable Symptoms

Patients with Hwabyung reported physical and psychological symptoms. The patients experienced physical symptoms, such as heat sensations, palpitations, and chest tightness. They also found it difficult to block excessive emotions and negative thoughts (30–32, 34). They appeared unstable and imperfect (31). During the recovery process, they are still sensitive and irritable to stimuli (34).

#### Category 2: Expressions of Anger

Most Hwabyung patients either expressed their anger through emotional outbursts or suppressed it, and they had difficulty changing this pattern and regulating their emotions. The manner in which anger was expressed seemed to be linked to personality traits (30, 31, 36).

#### Category 3: Perspectives on Recovery

The patient's perspective on recovery from the disorder was affected by the extent to which they blamed the external environment and their attitude toward self-regulation. Patients who thought the effects of external stress were absolute seemed to show a passive and pessimistic attitude. On the other hand, patients who thought self-regulation was important showed a relatively active attitude. However, they also had the limitation of avoiding direct problem-solving (34, 36). Patients demonstrated different expectations regarding professional help, especially psychotherapy, based on their willingness to change. Some looked forward to the effects of counseling, but others thought that Hwabyung had no therapeutic options, or that treatment was useless because symptoms recurred even after counseling sessions (36). Overall, the patients were less confident about controllability.

### Synthesized Finding 4: Compromise and Temporary Coping

**Figure 5:**
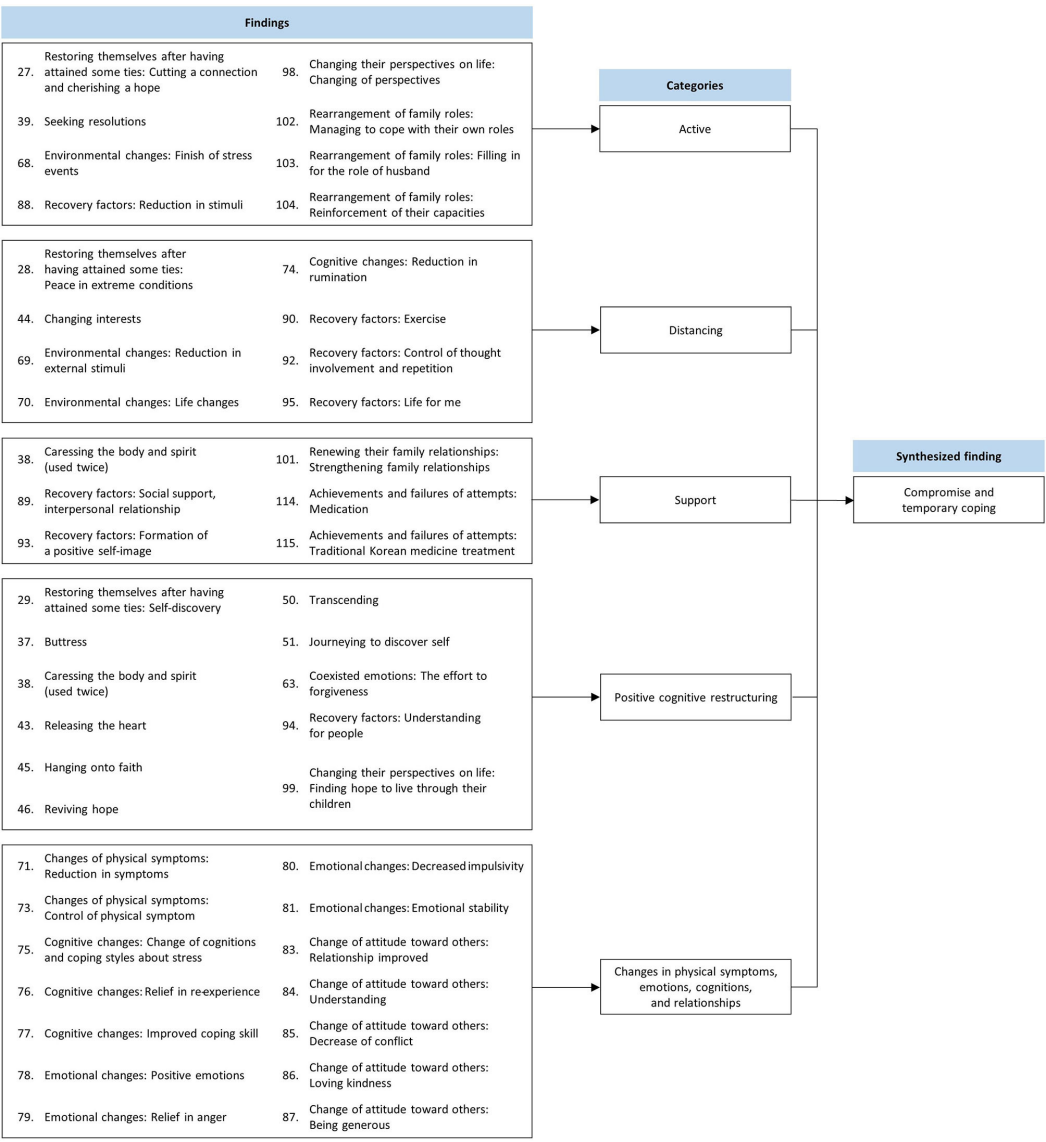
Synthesized finding 4: Compromise and temporary coping.

This synthesis was based on 47 findings aggregated into three categories (Figure 5). Generally, coping strategies are categorized as: problem-focused (active), emotional, social, and meaning-focused (41). During stressful daily life, Hwabyung patients tried to compromise with their affliction (e.g., abusive partner, financial difficulties, or problem children). They sometimes used active coping strategies to protect themselves or maintain their values. If the coping strategies were adaptive, the patients experienced improvements in physical symptoms, emotions, cognitions, and relationships. If not, they would attain only temporary relief.

#### Category 1: Active

Specific active coping strategies differed according to the source of distress. For example, Hwabyung patients who suffered from domestic abuse divorced their husbands. Some have sought new solutions to resolve their conflicts. Patients who needed to make life changes took on this responsibility and worked hard to improve their situations and, consequently, their symptoms (31, 32, 34, 35).

#### Category 2: Distancing

Distancing is an emotion-focused coping strategy. Hwabyung patients commonly used distancing as a coping strategy because they believed that nothing could be done to improve their symptoms (31, 32, 34).

#### Category 3: Support

Support coping includes all efforts to seek emotional and instrumental support (41). Hwabyung patients experienced improvement when they received support, encouragement, and comfort from family, friends, or neighbors. It was also found that taking medication or seeing a doctor was helpful in relieving symptoms (32, 34–36).

#### Category 4: Positive Cognitive Restructuring

Positive cognitive restructuring is a meaning-focused strategy. Some Hwabyung patients focused on the positive aspects of their lives and themselves (31, 32). Some developed new hopes or goals (32, 35) or chose to undergo a process of loving kindness (e.g., understanding, acceptance, and forgiveness) (32–34). In other cases, they found support through religion (32).

#### Category 5: Changes in Physical Symptoms, Emotions, Cognition, and Relationships

Patients who recovered from Hwabyung recognized improvements in various aspects of their lives. Physical symptoms decreased. Emotions can be regulated and stabilized, even when patients are confronted with the same stressors experienced before recovery. Negative thoughts turned into positive. Interpersonal conflicts subsided, and the relationships improved (34).

## Discussion

### Principal Findings

In this review, we aggregated qualitative evidence on the experiences and perspectives of Hwabyung patients and developed four synthesized findings: (i) anger arousal, (ii) blame, (iii) uncontrollable physical and emotional symptoms, and (iv) compromise and temporary coping. These syntheses showed patients' experiences throughout the course of the disorder and how they handled their distress and symptoms. Moreover, this review provides further evidence that Hwabyung is a mental disorder that represents complex symptoms of chronic anger, including physical symptoms and changes in thought, emotion, behavior, and social interaction.

The first synthesized finding, “anger arousal,” indicated the reasons Hwabyung patients felt anger. The development of Hwabyung is mainly related to interpersonal or family conflicts and other social issues (e.g., poverty, economic damage, and discrimination in the social networks). Anger is one of the victims' responses in interpersonal conflicts (42) and aims to change the opponent's behavior through attack or retaliation (43). Hwabyung patients perceive that their situation is unfair, so their emotions approximate resentment, which is a hostility against injustice (44). Additionally, because anger is a basic emotion toward a perceived threat to one's safety (45), life-threatening events such as disasters (46) and fatal diseases such as cancer (47) can be causes of Hwabyung. Tuomisto and Roche (48) also argued that traumatic events may evoke anger-related responses as well as anxiety and fear. Lastly, frustration (especially disappointment in children) also triggers anger for Hwabyung patients, and, according to previous studies (44, 49), this implies that Hwabyung patients perceived the failure of their children as a wrong that thwarted their beliefs and values.

The second synthesized finding, “blame,” indicated that the experiences of Hwabyung patients were complex and ambivalent. This theme includes inappropriate regulation strategies, such as suppression and rumination. Suppression cannot decrease anger, and rumination worsens anger (50). If the patients had failed to solve their problems despite their endurance, they indiscriminately blamed themselves, others, their circumstances, and fate. A recent study revealed that the belief that the world is unfair to oneself and the tendency to blame others are both associated with Hwabyung (51). Similarly, mourners who grieve unnatural deaths (i.e., accidents, suicides, or murders) can use a complex attributional strategy of dual blame; self-blame can damage self-esteem directly, and the desire for revenge can affect an individual's emotions and keep them focused on their loss (52). Although considering the many possible causes of a situation may be an attempt to better understand and control it, “self-blame” and “other-blame with desire for revenge” can hinder recovery. Dual blame negatively affects the thoughts, feelings, and behaviors of Hwabyung patients.

The third synthesized finding “uncontrollable physical and emotional symptoms” indicated that Hwabyung patients had difficulty controlling their symptoms and emotional expressions. Hwabyung patients felt that their condition had already exceeded the level at which it could be controlled. In fact, they were in a difficult state, in which they could no longer manage not only external stress but also themselves. For instance, they became more sensitive to external stimuli, they could not control their emotional changes, and they experienced physical symptoms. In addition, patients had two different perspectives regarding their expressions of anger and recovery. Patients' expressions of anger can be classified into two types: suppressed vs. explosive. Those who burst out of anger said that if they could hold back their anger, the problem would be solved, whereas those who could tolerate anger said that persevering in this way was unlikely to improve their problem. However, they said that they could not easily change the way they expressed anger. The belief that emotions are uncontrollable reduces the likelihood of using effective coping strategies such as cognitive reappraisal, which creates a vicious cycle (53). Thus, patients might plan to maintain their situation in the belief that they were born with a specific personality and temperament.

The fourth synthesized finding, “compromise and temporary coping,” indicated that patients tried various coping strategies to relieve their distress in a difficult situation where they had to choose between their own safety and values. For instance, issues related to domestic violence can be addressed by leaving the home in a safer environment. However, most Hwabyung patients live with daily stress to uphold traditional values or to protect their children. For this reason, these patients mainly use emotion-focused coping strategies such as distancing, emotional social support, and cognitive reappraisal, and receive professional support for uncontrollable physical symptoms. These patterns were also observed in other survivors of domestic violence (54). If a response strategy is successful, changes in various aspects of the patient's life can be observed, along with psychological growth, which is an improvement over the patient's condition before the stressful event (55).

### Issues Raised in Hwabyung

#### Gender Differences, Culture, and Hwabyung

According to earlier studies, women are more vulnerable to Hwabyung than men. The prevalence of Hwabyung in females was almost 2.5 or 3 times higher than in males (males: 2.1–2.5% and females: 5.6–7.5%) (11, 14). As with other mental disorders, including depression and anxiety disorders, reasons for gender differences in the prevalence of Hwabyung are not yet clear. Potential underlying biological factors are sex differences in hypothalamic-pituitary-adrenal (HPA) axis responses to stress (56), reproductive hormones (57), neuro-immune system (58) brain structure and function (59), and epigenetic environment (60).

At the same time, women' s social vulnerability of women exists in a cultural context (61). As cultures influence individual's beliefs, values, and behavioral patterns, Hwabyung has been considered a specific syndrome found in Koreans or Korean immigrants (4, 9, 21, 25, 31, 62–64). In Korea, which has a collectivistic and patriarchal society, women are particularly vulnerable to an unfair treatment, including domestic abuse and being forced to sacrifice for their families, especially their children. Nevertheless, Korean women believe that taking care of their families and raising children well is a task given to them as members of society. The self-esteem of Korean women depends on their children (65).

However, notably, from the perspective of female Hwabyung patients, the low marital satisfaction, a risk factor for Hwabyung, is significantly related to the husband's unfair attitude toward the role of women rather than the perception of the wife's gender role (61). This conclusion suggests that the spouses' attitudes are a more critical factor in the development of Hwabyung than the internalized cultural values of the patients.

In summary, we believe that both genetic and environmental influences are correlated to vulnerability of women to Hwabyung. Although Hwabyung was only investigated in Korea, we believe that these underlying experiences may extend beyond Korean culture, because this review focused on individuals' psychological experiences. Because these assumptions are not yet supported by empirical evidence, further studies on Hwabyung will be required to investigate the distinction between emic and etic manifestations of Hwabyung.

#### Stress and Hwabyung

Horowitz (66) argued that stress reactions, such as grief, adjustment disorder, and posttraumatic stress disorder, correspond to stress response syndromes. However, Simmen-Janevska and Maercker (67) proposed a model of normal and pathological stress reactions by modifying the Horowitz stress-response model (68). They differentiated pathological symptoms, dysfunctional behavior (e.g., abusing tranquillisers), and preoccupations, from normal responses such as alternating suppression/denial and intrusions. A normal stress response usually results in adaptation and relative completion, while a pathological consequence is a mental disorder or personality change. In other words, what Simmen-Janevska and Maercker's model (67) implies is that the difference between “adaptive” and “pathologic” response is the intensity of distress applied to life.

Bonanno (69) described four trajectories of potentially traumatic events (e.g., loss and trauma): chronic, delayed, recovery, and resilience. A recent review of trajectory models (55) has provided evidence that various consequences appear after traumatic stress. According to the review, most people maintain or recover their mental health even after a traumatic event. In contrast, a small percentage of people progress chronically (delayed onset in 8.9% of the population and chronicity in 10.6%). Although resilience is a natural response to traumatic events and occurs in the majority of those who have suffered such an event, people use various coping strategies and have different outcomes. Therefore, we need to go beyond the dichotomy of normal and abnormal and investigate the details of adaptation processes and mechanisms (69).

At the beginning of Hwabyung research, a survey of Korean psychiatrists and doctors of traditional Korean medicine revealed that the general population considered Hwabyung synonymous with “neurotic” or “stress” related conditions (70). However, during the next two decades, the experts gradually distinguished Hwabyung from stress response and established it as a mental disorder because it presented distinct emotional and physical symptoms and the patients complained of serious distress (25).

Several empirical studies have likewise indicated that the severity of psychiatric symptoms in Hwabyung is clinically meaningful. A previous cross-sectional study (18) reported mean scores of Center for Epidemiologic Studies Depression (CES-D) scale in Hwabyung group (*n* = 92) was 31.98 points, which indicated clinical depression. Additionally, state anger scores of STAXI in Hwabyung group were significantly higher (16.71 points) than that of non-Hwabyung group (13.43 points). A recent study (71) also found that Hwabyung patients (*n* = 58) scored significantly higher for depression, anger, hostility, and aggression than patients with clinical depression (*n* = 180). Another study (72) reported slightly different results, but this inconsistency may be due to small samples of Hwabyung group; the mean scores of STAXI were significantly higher in Hwabyung group (*n* = 6) than in the non-Hwabyung group (*n* = 283), 52.83 points vs. 39.8 points, but there was no significant difference in depression scores between the two groups.

We believe that the consequence (i.e., severity and chronicity of symptoms) determines whether a response to stress is pathological or adaptive. Therefore, we consider Hwabyung to be a mental disorder rather than an adaptive response.

#### Comparison of Hwabyung and Other DSM Disorders

In the DSM-5 (73), two diagnoses mainly focus on recurrent aggressive outbursts: intermittent explosive disorder (IED) and disruptive mood dysregulation disorder (DMDD). IED and DMDD share core features (temper outbursts and behavioral outbursts), but there are some differences. For instance, IED is characterized by behavioral outbursts that occur twice weekly, whereas DMDD comprises three or more temper outbursts per week and persistent irritability or anger, which begins in adolescence (with patients under 10 years of age) and cannot be diagnosed for the first time in adults (over 18 years). The persistent anger present in DMDD is diagnostically and clinically the most important difference between IED and DMDD. That is, IED patients spend less time in an angry state between behavioral outbursts (74). According to DSM-5 (73), the risk factors of IED include a history of physical and emotional trauma, serotonergic abnormalities, and amygdala hyperactivity to anger stimuli, while those of DMDD involve temperament and deficits in information processing and attention to emotional stimuli.

Compared to IED and DMDD, anger in the major diagnostic criteria of Hwabyung is inner experience (i.e., feeling of unfairness and subjective anger) and not aggression (19). Although this review included the subthemes of “tantrum” (31), “quick-tempered personality” (30), “volatile type,” and “volatile personality traits” (36), these indicate part of a complex of emotions, and are kinds of specifiers. The synthesized finding of “blame” indicates that Hwabyung patients experience various emotions as well as anger, and that this involves inward or passive aggression, rather than behavioral outbursts. Another synthesized finding of “uncontrollable physical and emotional symptoms” addresses the outcome that Hwabyung causes not only psychological but also physical symptoms. Physical symptoms, such as respiratory stuffiness, heat sensation, feeling of something pushing up, and epigastric mass, are extensively emphasized in the diagnostic criteria of Hwabyung (19). Moreover, in terms of etiology and risk factors, Hwabyung symptoms develop and progress during or after extremely stressful events, such as domestic violence or financial problems, in which anger may have been suppressed, so the onset of Hwabyung frequently occurs in middle-aged or later life (18). The synthesized finding of “anger arousal” also showed that anger triggers in Hwabyung were not episodic but long-term stressors, unlike in IED and DMDD.

As mentioned in the introduction, Asians commonly suppress their emotions. Several papers (75–77) have suggested that Asians complain more about physical symptoms than psychological symptoms in mental disorders. Thus, many researchers have considered Hwabyung to be a subtype of depression in Korea (78–80). Some researchers have described that anger and depression appear sequentially in the progression of Hwabyung, such as in a cyclothymic disorder (81). Although there are differences between these studies, depression, including major depressive disorder (MDD) and dysthymic disorder, was found in 30.5–37.5% of Hwabyung patients (18, 82). In one study (83), 60.7% of Hwabyung patients had underlying MDD. Despite the high comorbidity, the two are not exactly the same, and one is not completely included in the other.

Compared to depression, Hwabyung is more somatic, hyperarousal, and severe with regard to anger (71, 84). Hwabyung researchers have paid attention to its distinctive physical symptoms and typical causes, as well as anger, in order to distinguish between Hwabyung and depression (39, 85–87). The physical symptoms of Hwabyung include respiratory stuffiness, a heat sensation, a feeling of pushing up in the chest, a feeling of a mass in the throat or epigastrium, dry mouth, headache or dizziness, palpitations, and sighing (19). These symptoms are well clustered in this syndrome. Another difference between Hwabyung and MDD is that Hwabyung must be preceded by a stressful event, and these events are typically related to family, close neighborhoods, or financial problems (see synthesized finding “anger arousal”). The patient resents and blames a particular object or situation (see the synthesized finding “blame”), whereas MDD patients feel guilty. It should also be noted that Hwabyung's diagnostic criteria do not include suicidal ideation (19).

Lastly, we should compare Hwabyung and adjustment disorder. According to DSM-5 (73), adjustment disorder is defined as the presence of emotional or behavioral symptoms in response to one or more identifiable stressors. In adjustment disorders, symptoms develop and then disappear in the short-term depending on the onset and end of the stressor. However, unlike adjustment disorder, Hwabyung symptoms last after the stressor or its consequences are terminated. As shown in the results of meta-aggregation, Hwabyung patients stated that they could not improve because they still remembered stressful events (34, 36). It has been consistently reported that persistent and aggravating factors of Hwabyung are not only stressors but also rumination of bad memories (88) and blame (51).

#### Necessity for New Anger Disorder

Hwabyung has been proposed as a new anger disorder, but it is currently not reflected in the DSM-5 (73). Min, a Korean psychiatrist, has consistently suggested that Hwabyung could offer the opportunity for the formulation of a new model of pathological anger (89) and proposed an anger disorder in 2008 (16). Following this proposal, we agree on the need for a new anger disorder in official classification systems such as the DSM and ICD.

Explosive and aggressive outbursts are not the only anger-related mental problems, and various anger-related symptoms or syndromes have been reported. For instance, Fava (90) first reported anger attacks in patients with MDD in 1990. Anger attacks are associated with persistent irritability in patients with MDD (91). Linden (92–94) introduced posttraumatic embitterment disorder (PTED) as a new subtype of adjustment disorder in 2003. PTED could occur because of negative life events, to which the patient with PTED experiences embitterment, rage, and helplessness, and reacts with emotional arousal. The characteristic symptoms of PTED are repeated intrusive memories and persistent negative changes in one's mental well-being. In addition, Ataques de Nervios (of Mexico) and Stagnation Syndrome (of China) also share some characteristics of Hwabyung, such as suppression and affective dysregulation. The experience of Hwabyung's “uncontrollable physical and emotional symptoms” is very similar to the core feature of Ataques de Nervios in Puerto Rico. A study exploring Ataques de Nervios also identified that patients experienced “loss of control” of their emotional expression, bodily sensations, behavior, and nervous system due to psychological shock during the illness period (95). On the other hand, Stagnation Syndrome is defined as “a general term for diseased states characterized by a depressed mood with feelings of despair or uneasiness” in the *International Standard Terminologies on Traditional Medicine* by the World Health Organization (96). Stagnation Syndrome includes depression, restlessness, emotional changes, lack of energy, frequent sighing, and poor appetite (97, 98). Leng et al. (99, 100) suggested that Stagnation Syndrome was related to MDD and functional somatic syndrome, because Stagnation Syndrome showed various bodily symptoms and psychological problems.

All of these reports have indicated that there are patients with persistent negative emotions and intermittent anger attacks, but without explosive and aggressive outbursts. It is necessary to explore the aspects of anger disorder caused by the suppression of anger in a variety of contexts and cultures, and to reconcile the findings to suggest a new concept of mental disorder. Research into various anger disorders, including Hwabyung, will help complement the current mental health system.

### Limitations

All of the participants in the included studies were women, which could be a limitation. In particular, many studies have been conducted on middle-aged women; therefore, caution must be exercised when applying the results of the present study to men or younger patients. It is thought that the studies were conducted only on women because the prevalence of Hwabyung among women is very high compared to men (11–14).

In addition, this review includes a number of graduate theses. Although these have not been peer-reviewed, we decided to include them if there were no issues in terms of quality because the patient's perspective is abundantly presented in these papers.

Finally, only one qualitative study included long-term follow-up, so it was not possible to understand in detail how patients' experiences changed over time during the course of their illness. However, because most of the studies included in this review reflected the patient's overall life experience, the results provide a better understanding of Hwabyung from onset to recovery.

### Clinical Implications

By exploring the characteristics of the disorder and meta-synthesis of patients' experiences and perspectives, we can define and identify Hwabyung. This study was conducted to understand Hwabyung patients and manage their distress, but it was not intended to stigmatize them as dysfunctional individuals.

According to our results, Hwabyung patients experience stressful events perceived as unjust and wrong, fail to resolve their anger or resentment, and consequently suppress the events and anger. Their distress and suffering occur during or persist after a stressful event. One of the core experiences of Hwabyung is the loss of control. Particularly, they have difficulties in emotional regulation, and it is necessary to improve their emotional expression in appropriate ways. Flexibility in emotional expressions using both suppression and enhancement is an indicator of resilience (101). Being flexible is very important to patients with various mental disorders (102, 103). Specifically, according to Southwick et al. (104), Bonanno focused on regulatory flexibility because it can be learned. When emotions are controlled well and resilience is improved, Hwabyung patients may expect a better condition than before.

### Conclusions

This review explores the experiences and perspectives of Hwabyung patients in Korea. Hwabyung is an anger disorder that occurs through complex cognitive processes, such as resentment and frustration toward oneself and others, and affects patients' physical, psychological, and social functions. Especially, Hwabyung patients have difficulties in regulating emotions. These results can enable health professionals to better understand patients' feelings of loss of control and their pessimistic perspectives on recovery. Under stressful situations, Hwabyung patients usually use emotion-focused coping strategies, such as “distancing” and “seeking social support,” but these strategies just temporarily relieve their distress. Therefore, professional support is required to promote better coping among patients with mental disorders. Considering that suppressed anger is not treated as a specific concept and a primary issue in the study of mental disorders, Hwabyung can be a representative model of chronic anger disorder.

## Data Availability Statement

The original contributions presented in the study are included in the article/Supplementary Material, further inquiries can be directed to the corresponding author/s.

## Author's Note

This review was described in the Ph.D. thesis of the first author (HWS) (105), but the search, main results, and discussion are updated for this paper.

## Author Contributions

HWS and KBL selected the studies and extracted the data. HWS aggregated the findings according to similarity in meaning and merged them into categories, and then SYC, MP, BHJ, and JWK reviewed the results. HWS wrote the first draft of this manuscript. SYC and JWK have critically revised the manuscript. All authors have read and approved the final manuscript.

## Conflict of Interest

The authors declare that the research was conducted in the absence of any commercial or financial relationships that could be construed as a potential conflict of interest.
