# Supplementary material for: How Suppressed Anger Can Become an Illness: A Qualitative Systematic Review of the Experiences and Perspectives of Hwabyung Patients in Korea
Source: Front Psychiatry. 2021 May 28;12:637029. doi: 10.3389/fpsyt.2021.637029 (PMC8195628; doi:10.3389/fpsyt.2021.637029)
Supplement: Supplementary file 1 [file Table_1.docx]

Supplementary Table 1. Search strategies for each database

|  | **Search strategies** |
| --- | --- |
| PubMed | #1. hwabyung[Title/Abstract]  #2. hwabyeong[Title/Abstract]  #3. hwa-byung[Title/Abstract]  #4. hwa-byeong[Title/Abstract]  #5. anger-disease*[Title/Abstract]  #6. anger-disorder*[Title/Abstract]  #7. anger-syndrome*[Title/Abstract]  #8. anger-illness*[Title/Abstract]  #9. fire-disease*[Title/Abstract]  #10. fire-disorder*[Title/Abstract]  #11. fire-syndrome*[Title/Abstract]  #12. fire-illness*[Title/Abstract] |
| EMBASE | #1. hwabyung:ab,ti,kw  #2. hwabyeong:ab,ti,kw  #3. hwa-byung:ab,ti,kw  #4. hwa-byeong:ab,ti,kw  #5. 'anger disease*':ab,ti,kw  #6. 'anger disorder*':ab,ti,kw  #7. 'anger syndrome*':ab,ti,kw  #8. 'anger illness*':ab,ti,kw  #9. 'fire disease*':ab,ti,kw  #10. 'fire disorder*':ab,ti,kw  #11. 'fire syndrome*':ab,ti,kw  #12. 'fire illness*':ab,ti,kw |
| AMED | “hwabyung” OR “hwa-byung” OR “hwabyeong” OR “hwa-byeong” OR "anger disease" OR "anger disorder" OR "anger syndrome" OR "anger illness" OR "fire disease" OR "fire disorder" OR "fire syndrome" OR "fire illness" |
| CINAHL | “hwabyung” OR “hwa-byung” OR “hwabyeong” OR “hwa-byeong” OR "anger disease" OR "anger disorder" OR "anger syndrome" OR "anger illness" OR "fire disease" OR "fire disorder" OR "fire syndrome" OR "fire illness" |
| PsycARTICLES | #1. AB,TI,IF(hwabyung or hwabyeong or hwa-byung or hwa-byeong)  #2. AB,TI,IF(anger-disease* or anger-disorder* or anger-syndrome* or anger-illness*)  #3. AB,TI,IF(fire-disease* or fire-disorder* or fire-syndrome* or fire-illness*) |
| KMbase | ((((([ALL=화병] OR [ALL=홧병]) OR [ALL=울화병]) OR [ALL=火病]) OR [ALL=hwabyung]) OR [ALL=hwabyeong]) NOT ([ALL=氣鬱化火]) |
| KISS | 전체: (화병 \| 홧병 \| 울화병 \| 火病 \| hwabyung \| hwabyeong) NOT vase |
| NDSL | 전체: (화병 \| 홧병 \| 울화병 \| 火病 \| hwabyung \| hwabyeong) !vase |
| OASIS | #1. 화병: (제목: 화병 OR 초록: 화병 OR 키워드: 화병)  #2. 홧병: (제목: 홧병 OR 초록: 홧병 OR 키워드: 홧병)  #3. 울화병: (제목: 울화병 OR 초록: 울화병 OR 키워드: 울화병)  #4. 火病: (제목: 火病 OR 초록: 火病 OR 키워드: 火病)  #5. hwabyung: (제목: hwabyung OR 초록: hwabyung OR 키워드: hwabyung)  #6. hwabyeong: (제목: hwabyeong OR 초록: hwabyeong OR 키워드: hwabyeong) |

AMED=Allied and Complementary Medicine Database; CINAHL=Cumulative Index to Nursing and Allied Health Literature; KMbase=Korean Medical Database; KISS=Korean Studies Information Service System; NDSL=National Digital Science Library; OASIS=Oriental Medicine Advanced Searching Integrated System.
